# Correction: Hybrid AI/physics pipeline for miniprotein binder prioritization: application to the BRD3 ET domain

**DOI:** 10.1039/d5cc90427g

**Published:** 2026-01-13

**Authors:** Jokent Gaza, Monica J. Roth, Gaetano T. Montelione, Alberto Perez

**Affiliations:** a Department of Chemistry, University of Florida Gainesville Florida 32611 USA perez@chem.ufl.edu; b Quantum Theory Project, University of Florida Gainesville Florida 32611 USA; c Department of Pharmacology, Rutgers-Robert Wood Johnson Medical School 675 Hoes Lane Rm 636 Piscataway NJ 08854 USA; d Center for Biotechnology and Interdisciplinary Sciences, Rensselaer Polytechnic Institute Troy New York 12180 USA; e Department of Chemistry and Chemical Biology, Rensselaer Polytechnic Institute Troy New York 12180 USA

## Abstract

Correction for ‘Hybrid AI/physics pipeline for miniprotein binder prioritization: application to the BRD3 ET domain’ by Jokent Gaza *et al.*, *Chem. Commun.*, 2025, **61**, 19028–19031, https://doi.org/10.1039/D5CC05032D.

The authors regret that the following funding acknowledgment was not included in the original article: “The authors thank the support from the National Institutes of Health grants R35-GM141818 (to G. T. M.), R35GM122518 (to M. J. R.), and R01-GM149646 (to A. P.).”

The Royal Society of Chemistry apologises for these errors and any consequent inconvenience to authors and readers.

